# Experimental evidence for behavioural cooling as a response to virus infection in an ectothermic vertebrate

**DOI:** 10.1186/s12915-026-02669-6

**Published:** 2026-07-02

**Authors:** Dávid Herczeg, Veronika Bókony, Gábor Herczeg, Dóra Holly, Andrea Kásler, János Ujszegi, Tibor Papp, Attila Hettyey

**Affiliations:** 1 Department of Evolutionary Ecology, Plant Protection Institute, HUN-REN Centre for Agricultural Research, Budapest, Hungary; 2 HUN-REN-ELTE-MTM Integrative Ecology Research Group, Budapest, Hungary; 3 Department of Systematic Zoology and Ecology, ELTE Eötvös Loránd University, Budapest, Hungary; 4 Doctoral School of Biology, Institute of Biology, ELTE Eötvös Loránd University, Budapest, Hungary; 5 Disease Ecology and Wildlife Health Research Team, HUN-REN Institute for Veterinary Medical Research, Budapest, Hungary

**Keywords:** Anti-pathogen behaviour, Behavioural plasticity, Frog Virus 3, Host–pathogen interaction, *Rana dalmatina*, Thermoregulatory behaviour

## Abstract

**Background:**

Behavioural plasticity may contribute to the ability of wild animals to survive disease outbreaks. In absence of endogen heat control, ectothermic animals adjust their body temperature behaviourally. While many studies reported behavioural fever, its opposite, behavioural cooling—when infected animals lower their body temperature by using cool microenvironments—remains poorly documented.

**Results:**

Here, we report the first experimental evidence of behavioural cooling in an ectothermic vertebrate as a response to pathogenic infection. We investigated thermoregulatory responses in tadpoles of the agile frog *Rana dalmatina*, a cool-adapted amphibian, following experimental infection with a ranavirus that can replicate well at high temperatures. Tadpoles were placed either in thermal gradients or homogeneously cool environments for 5 days post-exposure. In thermal gradients, all tadpoles reduced their preferred temperatures over time, but this decrease was steeper in infected tadpoles, and individuals with higher infection intensities preferred cooler temperatures. Infected tadpoles decreased the range of their preferred body temperatures over time, while a similar trend was not detectable in non-infected tadpoles. Infection prevalence was similar between the two thermal environments, yet infection intensities were significantly higher in the thermal gradient.

**Conclusions:**

These results suggest fine-tuned thermoregulation by infected tadpoles to balance out the benefits of behavioural cooling for fighting a pathogen versus the immune-suppressive and developmental costs of low temperatures.

**Supplementary Information:**

The online version contains supplementary material available at https://doi.org/10.1186/s12915-026-02669-6.

## Background

Behavioural thermoregulation is the use of specific behaviours by an ectothermic organism to regulate its body temperature, allowing it to maintain thermal balance within a range that supports optimal physiological performance [1, 2]. Accordingly, behavioural thermoregulation can also shape the outcome of host–pathogen interactions [3]. Ectothermic hosts can respond to pathogen presence by displaying behavioural fever [4], i.e. attaining higher body temperatures by using warmer microhabitats after pathogen exposure [5, 6]. This way animals can create conditions more favourable for themselves than for the pathogen thanks to an enhanced immune function or by taking advantage of a thermal optimum mismatch between the pathogen and its host [5, 7–11]. Infections can induce behavioural fever in ectotherms [5, 6]. Diverse ranges of pathogens, including viruses, have been shown to trigger behavioural fever in amphibians [12–15], a group acutely threatened by disease pandemics and extinction risk worldwide [16].

Ranaviruses (hereafter *Rv*), belonging to the *Iridoviridae* family, evolved to infect a wide range of ectothermic vertebrates, causing ranavirosis [17]. Although ranavirosis alone cannot be held responsible for amphibian declines on a global scale, local epidemics have led to mass mortality events and, in some cases, resulted in the local extirpation of populations [18–20]. Furthermore, the widespread distribution [21] and broad host range [21, 22] of *Rv*s, coupled with their capability of interclass transmission between ectothermic vertebrates [23, 24], as well as the devastating outcomes of co-infections involving several *Rv*s or an *Rv* along with pathogenic bacteria or fungi [25] altogether confer *Rv*s dangerous potential for inducing massive collapses of amphibian populations on a global scale.

Temperature is the abiotic factor with perhaps the largest influence on the disease dynamics of ranavirosis in amphibians [26]. Attempts to determine the thermal optimum range of *Rv* delivered contradictory results. Under in vitro conditions, the *Rv* strain Frog Virus 3 (FV3) replicates successfully between 8 and 30 °C, with a lower replication rate below 15 °C and the highest at 30 °C [27, 28]. This thermal dependency of *Rv* propagation is supported by the observation that *Rv*-related mortality is most frequent during the warm summer months in wild amphibian populations [17]. Additionally, increasing the air temperature from 20 to 27 °C was shown to enhance *Rv* propagation, disease incidence, and mortality rates in the common frog *Rana temporaria* [26]. Also, *Rv*-infected common frog tadpoles suffered higher mortality at 20 °C compared to 15 °C [29]. Similarly, tadpoles of four amphibian species exposed to FV3 at 25 °C were consistently more likely to become infected and die than conspecifics exposed at 10 °C [30]. In contrast, *Rv* infection probability and mortality were lower at 22 compared to 14 °C in two *Lithobates* frog species infected with three different FV3 strains [31]. Moreover, in larvae of the agile frog *R. dalmatina*, exposure to 30 °C for 6 days resulted in reduced infection intensity of FV3, accompanied by enhanced host survival, as compared to conspecifics exposed to either 28 or 22 °C [32]. Finally, worth to note that even slight temperature changes can strongly influence the outcome of FV3 infections: a 2 °C increase (from 10 to 12 °C) in wood frog *Lithobates sylvaticus* larvae turned apparently persistent infections into lethal [30]. While these studies together highlight the effect of temperature on the progression of ranavirosis, inconsistencies in the observed patterns may stem from interspecific differences in the temperature-dependence of amphibian immune functions, in the temperature optima of *Rv* strains, or from varied outcomes of interactions between pathogen genotype, host genotype, and temperature [31]. It is important to point out that in all aforementioned studies, environmental temperatures were set by the researchers, so that the tested amphibians did not have the opportunity to exert their thermal preferences and adjust their body temperatures via behavioural thermoregulation. The only study we know of that provided *Rv*-infected amphibians with the opportunity to thermoregulate reported that metamorphic and adult *Anaxyrus terrestris* toads displayed behavioural fever. More specifically, metamorphic individuals exposed to *Rv* increased their preferred temperature by 3.52 °C 1 day post-exposure, while adults exhibited a 1.43 °C increase in preferred temperature 2 days post-exposure as compared to non-infected controls [12].

Here, we investigate whether larval agile frogs adjust their thermal preferences following exposure to *Rv*, and whether the opportunity for thermoregulation can reduce infection prevalence and intensity. To achieve these objectives, we placed *Rv*-exposed and *Rv*-unexposed tadpoles into homogeneously cool environment or into thermal gradients for 5 days, recorded their thermoregulatory behaviour, and measured infection intensities at termination. This setup allowed us to (i) study the thermoregulatory behaviour and temperature optimum of *Rv*-unexposed tadpoles, (ii) detect potential infection-induced changes in thermoregulatory behaviour, (iii) relate infection intensities to the magnitude of adjustments in thermoregulatory behaviour, (iv) observe potential changes in general movement activity in parallel to disease progression, and (v) assess the effects of a homogeneously cool vs. variable thermal environment on virus replication. Based on previous findings of lower *Rv* infection intensities coupled with higher survival at high temperatures in agile frog tadpoles [32], we expected behavioural fever in response to *Rv* infection. We predicted that infected tadpoles would prefer higher body temperatures than their healthy conspecifics in the thermal gradient, and that infected tadpoles with higher infection intensities will prefer higher temperatures. Additionally, we predicted activity of infected animals to decrease over time, with lethargy emerging as the disease progressed [33], and to observe a steeper decline in activity in the homogeneously cool environment (where high activity does not deliver obvious benefits) than in the thermal gradient (where behavioural thermoregulation may require moving around). We also expected infection intensities to be higher in the homogeneously cool environment as compared to the thermal gradient that had higher mean temperature and provided opportunity for thermoregulation (i.e. to express behavioural fever).

## Results

### Preferred body temperatures (T_pref_)

**Fig. 1:**
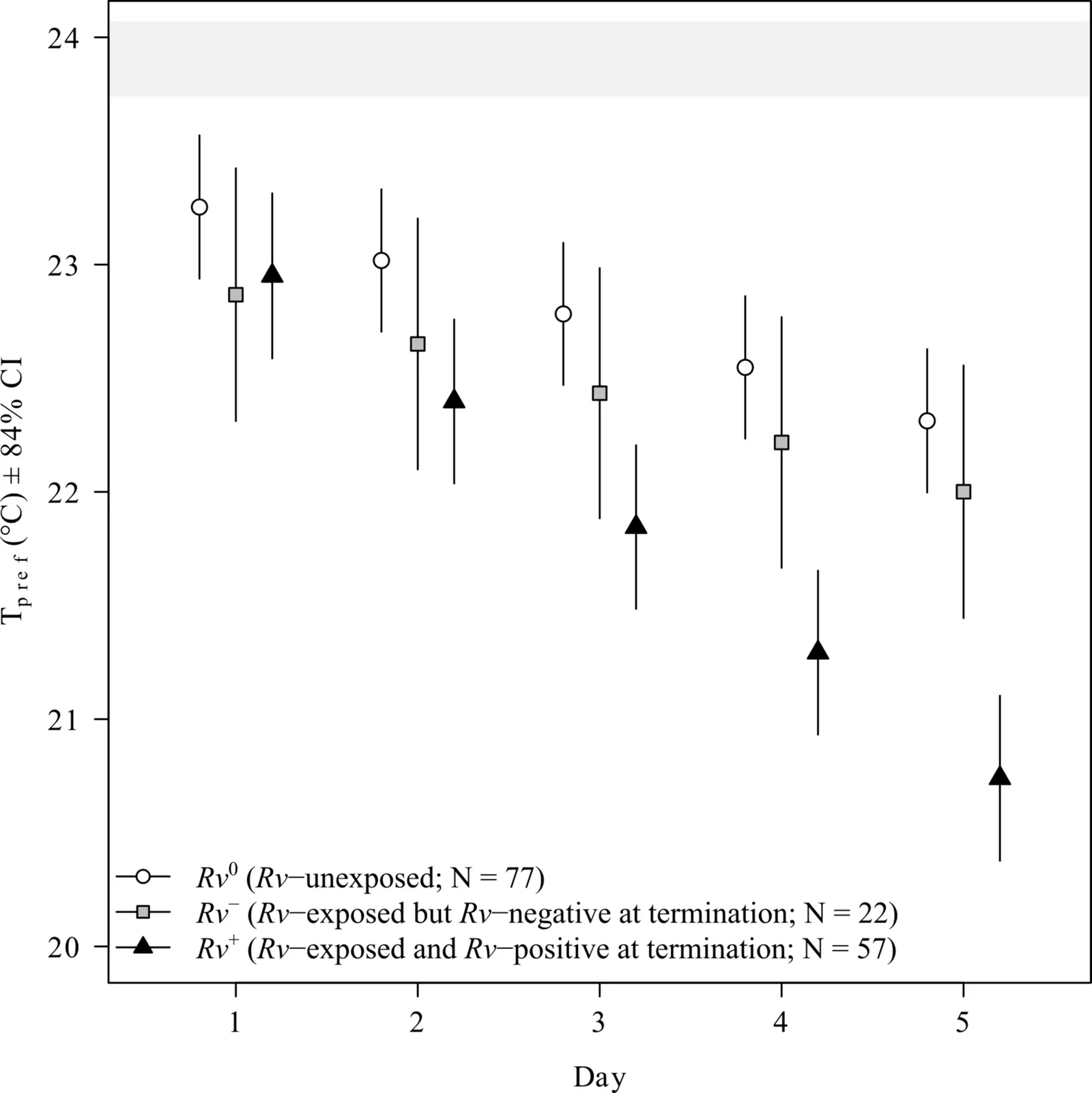
Preferred body temperatures (T_pref_) of agile frog tadpoles during the 5 days of the experiment. Means ± 84% confidence intervals (CIs) are shown, as estimated from the statistical models presented in Table 1. The grey horizontal bar shows the 84% CI of the mean of the temperatures available in the experimental arenas, representing the mean body temperature of hypothetical thermoconformer tadpoles that move randomly in the thermal gradient. T_pref_ CIs that do not overlap with the grey area indicate active behavioural thermoregulation, i.e. non-random choice of thermal environment

**Fig. 2:**
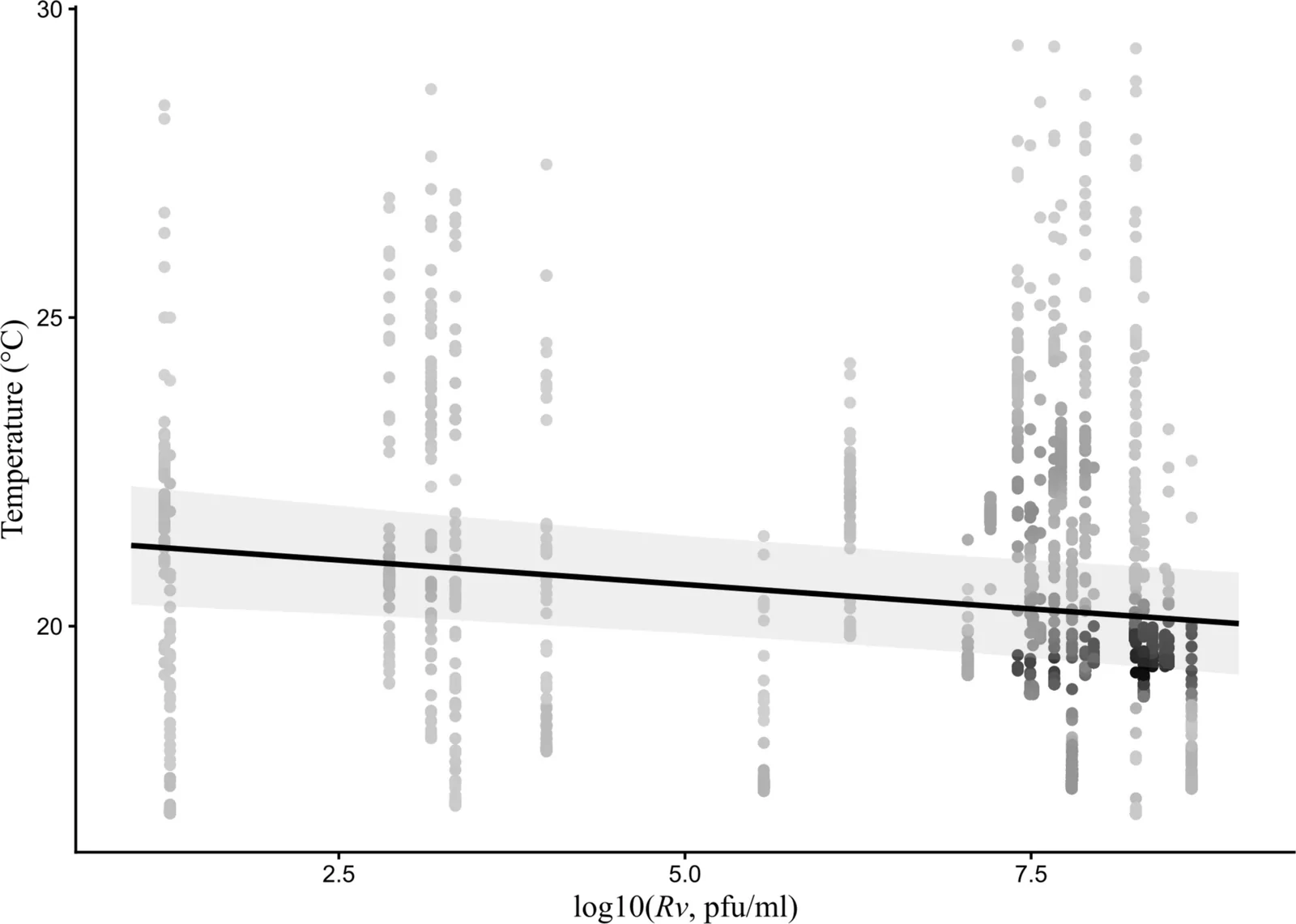
Temperatures selected in the gradients by *Rv*-positive tadpoles on the 5th day, in relation to their infection intensity. The black line and grey stripe, respectively, represent the regression line and its 95% confidence interval as estimated from the model in Table 1. Symbol darkness reflects local density: darker shade indicates a higher number of overlapping points

Across the 5 days, tadpoles in all treatment groups combined selected relatively high temperatures in the thermal gradient (22.4 °C ± 0.12 SE), both compared to the homogeneously cool environment (17.5 °C ± 0.12 SE; *t*_137_ = 33.66, *P* < 0.001; Additional file 1: Fig. S1A) and also compared to the average of the temperatures available in the thermal gradients (see non-overlapping CIs in Fig. 1). The T_pref_ of tadpoles in the thermal gradient was significantly affected by the two-way interaction of *Rv*-status and the number of days since exposure (Table 1, Fig. 1). The T_pref_ significantly declined in all treatment groups over time during the experiment (Additional file 1: Table S1), where the decrease was significantly steeper in the *Rv*^+^ group (i.e. in tadpoles that were exposed to *Rv* and were *Rv* positive at termination) compared to the *Rv*^−^ group and the *Rv*^0^ group, while there was no difference between the latter two (Additional file 1: Table S2; Fig. 1). As a result, by the 5th day, T_pref_ was significantly lower in the in *Rv*^+^ group compared to the other two groups, and already from the 3rd day compared to the *Rv*^0^ group (Fig. 1). The T_pref_ of *Rv*^0^ and *Rv*^−^ groups were not significantly different on either day (all *P* > 0.367). We found a significant negative effect of *Rv* infection intensity on the T_pref_ of *Rv*^+^ tadpoles (Table 1), i.e. tadpoles with higher *Rv* infection intensity had lower T_pref_ (Fig. 2).

**Table 1:** Type-2 analysis-of-deviance tables (Wald *χ*^2^ test) of the statistical models. Significant effects (*P* < 0.05) are highlighted in bold. Prevalence at termination expressed as the proportion of *Rv*-exposed and *Rv*-positive (i.e. *Rv*^+^) tadpoles at termination to tadpoles in the *Rv*-exposed infection group

Dependent variable	Predictors	*χ*^2^	df	*P*
Prevalence	Thermal environment	0.006	1	0.935
	Round of experiment	4.599	1	**0.031**
Infection intensity	Thermal environment	7.870	1	**0.005**
	Round of experiment	0.028	1	0.865
T_pref_	*Rv*-status	9.465	2	**0.008**
	Day	1092.26	1	** < 0.001**
	Round of experiment	4.315	1	**0.037**
	*Rv*-status × Day	216.452	2	** < 0.001**
T_IQR_	*Rv*-status	2.271	2	0.321
	Day	0.407	1	0.523
	Round of experiment	4.612	1	**0.031**
	*Rv*-status × Day	6.181	2	**0.045**
Activity	*Rv*-status	1.850	2	0.396
	Day	1.519	1	0.217
	Thermal environment	1.030	1	0.310
	*Rv*-status × Day	229.938	2	** < 0.001**
	*Rv*-status × Thermal environment	0.195	2	0.906
	Day × Thermal environment	9.756	1	**0.002**
	*Rv*-status × Day × Thermal environment	22.756	2	** < 0.001**
T_pref_ on day 5 in *Rv*^+^ individuals	log (*Rv*)	8.448	1	**0.003**
	Round of experiment	3.152	1	0.075
Activity on day 5 in *Rv*^+^ individuals	log (*Rv*)	1.712	1	0.190
	Thermal environment	1.167	1	0.682
	Round of experiment	0.669	1	0.413
	log (*Rv*) × Thermal environment	2.869	1	0.090

*T*_*pref*_ preferred body temperature, *T*_*IQR*_ interquartile range of temperatures selected over a day, *Rv-status *infection status on day 5 (*Rv*^0^: *Rv*-unexposed; *Rv*^−^: *Rv*-exposed but *Rv*-negative at termination; *Rv*^+^: *Rv*-exposed and *Rv*-positive at termination), *Day *the number of days since exposure, *log (Rv) *natural log-transformed ranavirus infection intensity

### Interquartile range of daily selected temperatures over a day (T_IQR_)

**Fig. 3:**
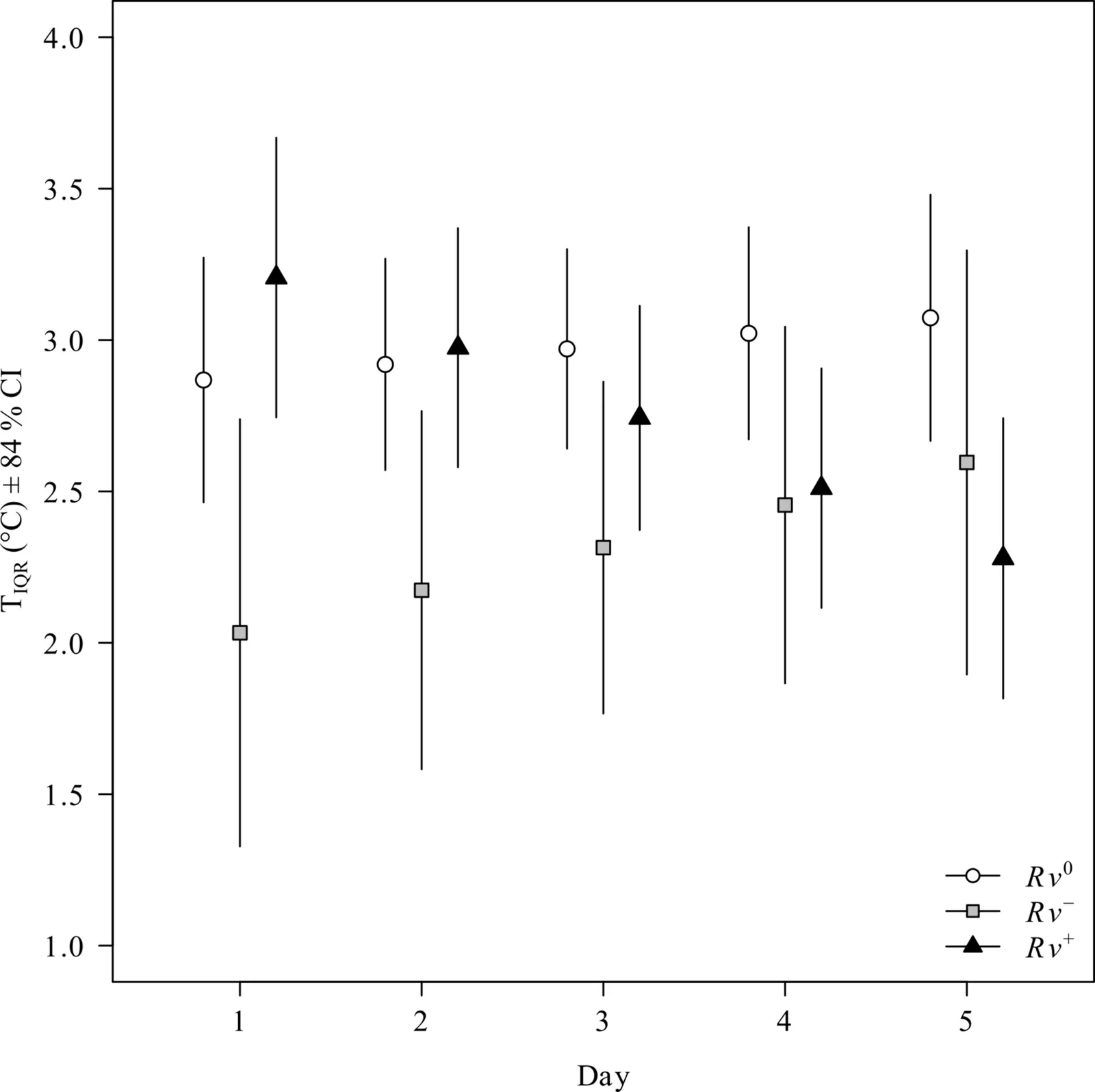
The interquartile range of temperatures selected over a day (T_IQR_) by agile frog tadpoles in thermal gradients. Means ± 84% confidence intervals (CIs) are shown, as estimated from the statistical models presented in Table 1

The T_IQR_ of tadpoles in the thermal gradient was significantly affected by the two-way interaction of *Rv*-status and the number of days since exposure (Table 1; Fig. 3). Specifically, T_IQR_ decreased over time in the *Rv*^+^ group, but not in the other two groups (Additional file 1: Table S1). However, there was no overall difference in the extent of T_IQR_ according to *Rv*-status (Additional file 1: Table S2), nor was there a significant difference on any 1 day (all *P* > 0.356).

### *Rv* prevalence and infection intensity

Five days post-exposure, 72.2% of *Rv*-exposed individuals had detectable *Rv* loads (*Rv*^+^) whereas 27.8% were *Rv*-negative (*Rv*^−^). Amongst the *Rv-*exposed individuals, infection prevalence did not differ between the thermal gradient (75.6%) and the homogeneously cool environment (76.5%, Table 1). However, infection intensity in *Rv*^+^ individuals was significantly higher in the thermal gradient (9.42 × 10^7^ pfu ml^−1^ ± 3.96 × 10^7^ SE) than in the homogeneously cool environment (2.79 × 10^6^ pfu ml^−1^ ± 1.12 × 10^6^ SE; incidence rate ratio: 33.7 ± 19.5 SE, *z* = 6.08, *P* < 0.001; Additional file 1: Fig. S1B).

### Movement activity

**Fig. 4:**
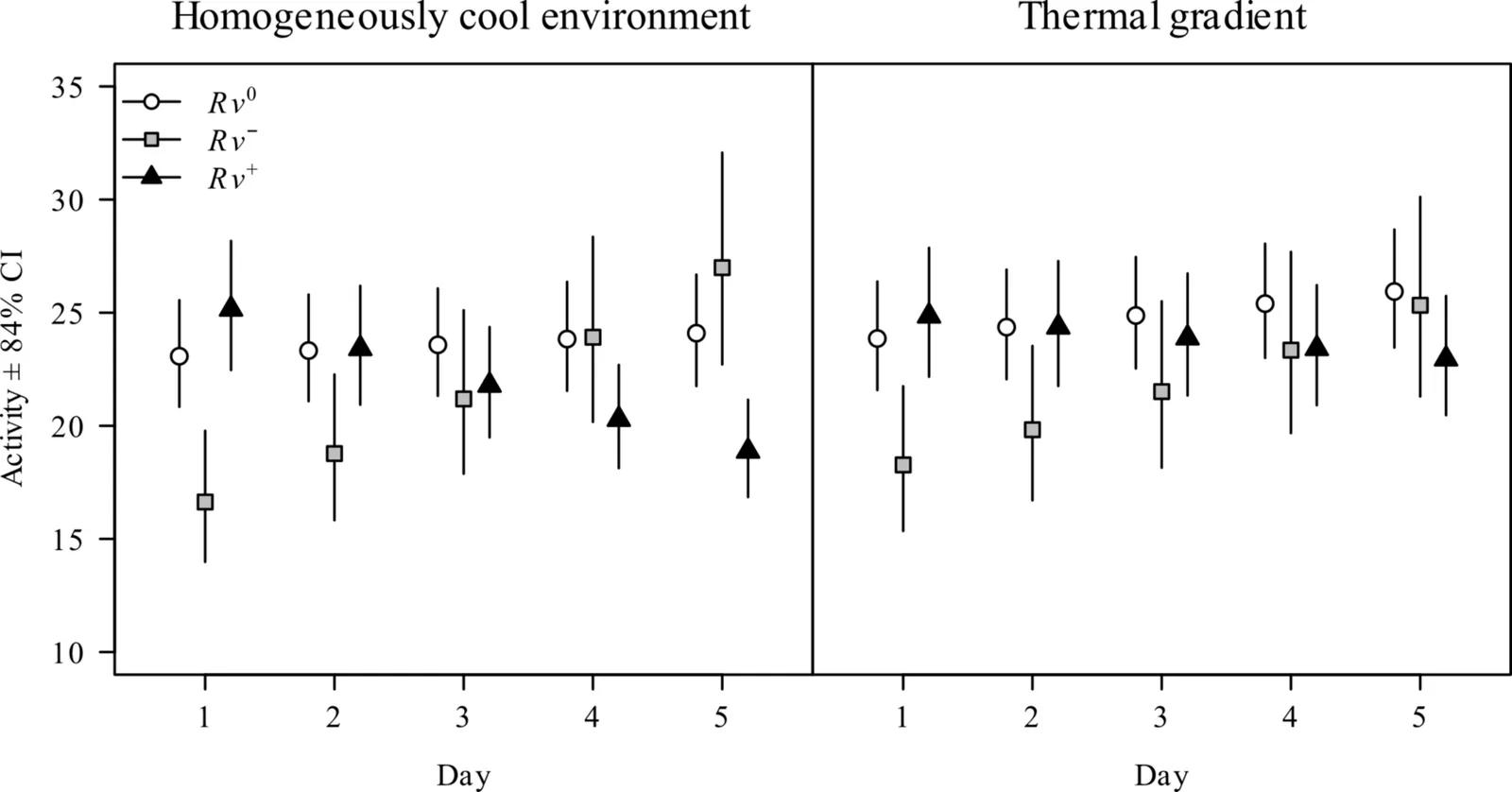
Activity of agile frog tadpoles during the experiment. Means ± 84% confidence intervals (CIs) are shown, as estimated from the statistical models presented in Table 1. Activity was estimated by measuring the distances between consecutive positions of tadpoles determined from recordings taken 2 min apart. Distance units were arbitrary but consistent across all video recordings. They were derived directly from the video recordings and were therefore not converted into SI units

The movement activity was significantly influenced by the three-way interaction of the type of thermal environment, *Rv*-status, and the number of days since exposure (Table 1; Additional file 1: Table S2; Fig. 4). The temporal change of activity varied across all treatment combinations except for *Rv*^0^ individuals between the two thermal environments (Additional file 1: Table S2, Fig. 4). Specifically, *Rv*^0^ tadpoles showed a slight increase in activity over time, but this was significant only in the thermal gradient; *Rv*^+^ tadpoles decreased activity with time in both environments, with a steeper decrease in the homogeneously cool environment; and *Rv*^−^ tadpoles exhibited increased activity over time in both environments, with a steeper increase in the homogeneously cool environment (Additional file 1: Table S2, Fig. 4). We found no effect of infection intensity on activity patterns on day 5 (Table 1). Finally, there was no significant difference in body mass between treatment groups (*χ*^2^ = 0.012, df = 2, *P* = 0.108, Additional file 1: Fig. S2).

## Discussion

The key finding of this study was that in the thermal gradient, infected tadpoles showed lower preferred body temperatures than non-infected conspecifics already a few days after infection. Simultaneously, infected tadpoles thermoregulated with increasing precision over time, while this change was not observable in healthy tadpoles. After 5 days of disease progression, infected tadpoles with higher infection intensity had lower preferred body temperatures. Infection intensity was close to two magnitudes higher in thermal gradients than in homogeneously cool environments, as tadpoles in the latter were kept at lower temperatures compared to the temperatures selected by tadpoles in thermal gradients. Finally, we detected decreasing activity in *Rv*-positive individuals in both environments, but the pattern was stronger in the homogeneously cool environment.

Adjustments in thermoregulatory behaviour in response to pathogens include behavioural fever, which has been documented in various invertebrates and ectothermic vertebrates, including amphibians [7, 8, 13–15, 34–37], even if its importance is debated, and the frequency of its occurrence across taxa is unknown [5, 38]. At the same time, an in vivo experiment suggested that exposure to high temperatures of around 30 °C can benefit agile frog tadpoles by reducing the severity of *Rv* infection [32]. Consequently, we expected agile frog tadpoles to show behavioural fever. Contrary to our predictions and previous results, we found that *Rv*-infected agile frog tadpoles displayed behavioural cooling from the third day after exposure to *Rv*. It is important to note that in the thermal gradient all animals displayed active thermoregulatory behaviour, as both unexposed and infected tadpoles spent most time at temperatures that were on average lower than the mean temperature of their thermal environment. Also, individuals with higher infection intensities displayed stronger behavioural cooling. At the same time, tadpoles in the homogeneously cool environment exhibited lower infection intensities than those in thermal gradients, although temperatures provided by the former could also be reached in the latter. Consequently, infected tadpoles displayed behavioural cooling by lowering their thermal preference compared to non-infected tadpoles, but not to the extent to maximize the anti-pathogen effect. This discrepancy may be explained by the contrasting needs and constraints for optimizing body temperature under *Rv* infection. While the immune system of the ectothermic hosts may work more efficiently at higher temperatures [39, 40], this beneficial effect may be offset by higher virus replication rates [26, 30] or by other costs associated with higher temperatures. Specifically, agile frog larvae experiencing high temperatures (28–30 °C) during larval development can suffer from increased mortality, delayed metamorphosis (without resulting in mass gain), and sex reversal [41]. Consequently, behavioural fever expressed upon infection with *Rv* may not pay off for this cool-adapted host species. However, this does not explain the surfacing of behavioural cooling.

Behavioural cooling, previously referred to as ‘behavioural chill’ or ‘cold-seeking behaviour’, was first documented in arthropods [42–46], subsequently also detected in rats [47] and iguanas [48], and was suggested to be a form of anti-pathogen behavioural defence. Similarly, ‘behavioural hypothermia’ has been reported in mice during bacterial infection [49], in lizards during tick infestation [50], and in amphibians as a beneficial response to hypoxia [51]. The evolution of behavioural cooling in agile frogs in response to FV3 may be explained by the capability of the virus to replicate under warmer temperatures, as in vitro studies have shown that FV3 reaches its highest titres at 30 °C [27, 28]. Consequently, low body temperature is likely to provide benefits to amphibian hosts because it presents unfavourable conditions for pathogen replication, while for cooling to be advantageous to hosts also requires that lower temperatures cause a slighter decrease in their immune function than in *Rv* replication. The benefit of low temperatures is supported by some experimental studies performed in vivo on other host species [26, 29]. Considering that the agile frog is a cool-adapted species, this aligns well with the notion that the relationship between temperature and the effectiveness of the immune response may differ amongst warm- vs. cool-adapted amphibians: The former may benefit from higher temperatures, while the latter may do best under cooler conditions [38, 52, 53], even if this relationship can be further complicated by additional environmental factors [11]. Whatever evolutionary force has led to its appearance, our study is the first to experimentally demonstrate pathogen-induced behavioural cooling in an ectothermic vertebrate.

Interestingly, infected tadpoles showing behavioural cooling in the thermal gradients did not target as low body temperatures as what their conspecifics experienced in the homogeneously cool environments and what resulted in lower infection intensity, even though such temperatures (and even lower) were available in the gradient. One explanation may be that their target of thermoregulation shifted due to a constant reassessment of the trade-off between the anti-pathogen benefits and the physiological costs of maintaining body temperatures below the optimum. Such suboptimal temperatures can reduce immune function [53, 54], metabolism, and locomotion [55, 56], and limit behaviours such as feeding, reproduction, and predator avoidance [56, 57]. The temporal dynamics of *Rv* infection is heavily influenced by the host’s immune system, which is largely shaped by ambient temperature during pathogen exposure and the infection dose [30, 32, 33, 58, 59]. For example, American bullfrog *Lithobates catesbeianus* tadpoles experienced exponential growth of *Rv* titres within 2 days post-exposure, with replication rates rapidly increasing by 1-week post-exposure, a point at which tadpole mortality began to occur [58]. It is also possible that if our experiment had lasted longer, infection intensities would have increased further, forcing infected tadpoles to choose even lower temperatures, further closing the gap between the temperature experienced in the homogeneously cool environments and T_pref_ in the thermal gradients. This is supported by the observation that T_pref_ decreased constantly from day to day and this curve has not yet levelled out by the 5th day. A gradual decline in preferred temperature over time may potentially occur in ectotherms exposed to persistently cool environments and could represent a compensatory acclimation response [60–62]. In such cases, individuals may initially select warmer temperatures to restore physiological performance but subsequently lower their preferred temperature as acclimation progresses. Similarly, Havird et al. [63] distinguished immediate passive thermal responses arising from *Q*_*10*_ effects (i.e. the factorial change in a biological rate associated with a 10 °C increase in temperature) from delayed active acclimation responses and demonstrated that biological rates can follow multiple trajectories through time. Thus, one alternative explanation for the temporal decline in T_pref_ observed in our experiment is that tadpoles gradually adjusted their thermal preference during acclimation to experimental conditions. However, this mechanism is unlikely to explain behavioural cooling in our study. Agile frog is a cold-adapted species that reproduce early in the year, and therefore eggs and early-stage tadpoles naturally develop under relatively cool conditions. Accordingly, tadpoles in our experiment were maintained at ~ 16 °C prior to the trials, corresponding to the mean water temperature experienced during larval development in May under natural conditions ([64]; Additional file 1: Fig. S5). Based on our experience, tadpoles reared under these conditions develop normally and remain healthy, suggesting that this temperature does not represent stressful cold exposure. Furthermore, 16 °C was available within the thermal gradient. Most importantly, compensatory acclimation alone cannot explain why *Rv*^+^ individuals exhibited a substantially steeper decline in T_pref_ over time compared to the other two groups. Therefore, the progressive reduction in T_pref_ more likely reflects a time-dependent behavioural response associated with disease progression rather than acclimation to experimental thermal conditions.

The determination of preferred body temperature, i.e. the body temperature voluntarily chosen in an ecological cost-free thermal gradient, is of central importance for understanding behavioural thermoregulation [65]. Studies exploring changes in behavioural thermoregulation as anti-pathogen behaviours focused so far on mean temperatures [12–15], even though thermal biology considers the ranges of selected body temperatures to be of central importance [65]. This is because thermoregulating animals typically do not aim to maintain one exact temperature value, but to keep their body temperature within a range. Therefore, approaching induced shifts in the target via estimating solely the mean, without considering the width of the range, is of limited use. Here, by showing that infected tadpoles did not only decrease the mean of their T_pref_ by 1.6 °C but also decreased the range of their preferred body temperature by ca. 1 °C, we demonstrate another layer of active behavioural cooling, suggesting that the importance of accurate thermoregulation increased following infection. Different physiological and/or biochemical processes have different thermal optima and sensitivity, and it was out of the scope of our study to determine these thermal parameters for both the host and the pathogen. However, considering the delicate balance of decreasing pathogen performance without too much loss of host performance in our host–pathogen system, the reported ca. 30% increase in precision during the 5 days of infection is very likely to be biologically significant. Interestingly, our finding contrasts with previous research demonstrating that malaria-infected side-blotched lizards exhibited less precise thermoregulation compared to their non-infected conspecifics [66]. However, this response may not be advantageous and could reflect parasite-induced alterations in host physiology and behaviour rather than an adaptive host strategy [66]. Thus, we encourage future studies on thermoregulatory responses to pathogens to incorporate both T_pref_ and T_IQR_ in their methodological framework.

According to our predictions, activity decreased over time in *Rv*^+^ tadpoles in both the homogeneously cool environment and the thermal gradient, with a steeper decrease observed in the former. This decrease in activity might not be explained by an automatic consequence of lower preferred temperatures, as activity also declined in the homogenously cool environment where the temperature remained stable. However, *Rv*-induced activity decrease was not detected within a comparable timeframe to ours in post-metamorphic individuals of either wood frogs *Lithobates sylvaticus* [67] or agile frogs [68], and has so far only been detected in the terminal stage of disease progression in tiger salamanders *Ambystoma tigrinum* [69]. In our case, the observed activity decreases in *Rv*^+^ tadpoles may have resulted from accumulating pathological damage or the energetic costs associated with an intensifying immune response during disease progression—most likely a combination of both. At constant lower temperatures applied in our experiment, immune responses may be less effective in amphibians [40], requiring more sustained metabolic effort to fight infections, thereby leaving less energy available for locomotion [70]. In contrast, within the thermal gradient, infected tadpoles may have used thermoregulation to optimize energy allocation more efficiently, allowing them to maintain higher levels of activity.

Curiously, we found increasing activity with time in the *Rv*^−^ tadpoles, both in the homogeneously cool environment and the thermal gradient, with a steeper increase observed in the former. This increase started from a low level of activity which gradually reached the level observed in the *Rv*^0^ group. It is unclear if in *Rv*^−^ tadpoles the experimental infection was successful but these individuals cleared *Rv* in 5 days or if experimental exposure to *Rv* was unsuccessful in this group of animals. The observed low activity immediately following exposure to *Rv* supports the former scenario. We speculate that these individuals exhibited a stronger/faster than average immune response to the pathogen, which in turn decreased their movement activity in the beginning. Ascertaining this hypothesis would, however, require targeted investigation.

## Conclusions

We detected behavioural cooling in an amphibian–virus host–pathogen system, where infected animals lowered their preferred temperatures by around 1.6 °C by the 5th day post-infection as compared to non-infected conspecifics. The strength of the behavioural response of agile frogs depended on infection intensity. Moreover, individuals maintained in a thermal gradient had roughly twice as high infection intensity than conspecifics maintained at the homogenously cool environment. This suggests that, at least within the 21–23 °C range, maintaining a lower body temperature in the presence of *Rv* may be advantageous to combat the infection. These two results together suggest that behavioural cooling might be an adaptive response to infection with *Rv*, but further experimental investigations will be necessary to prove this. Our observation that infected tadpoles decreased their target body temperature and at the same time increased their thermoregulatory precision draws attention to the fact that studies concerned with disease-induced changes in thermoregulatory behaviour should not only focus on the widely used T_pref_ but should also assess T_IQR_ to achieve a deeper understanding. In addition, we detected decreasing activity in *Rv*-infected tadpoles, which we interpret as a sign of progressing disease. We note that a longer observation period could have provided further information on how far behavioural cooling, infection intensity, and decreasing activity would have progressed. However, with longer observation periods, it becomes difficult to disentangle whether behaviour shapes infection status or, conversely, infection shapes behaviour. We speculate that both mechanisms act reciprocally. Under natural circumstances, behavioural cooling may manifest in seeking shaded areas and deeper water, or daily activity shifted towards cooler parts of the day. Such behavioural changes may, however, pose fitness costs, which may be exacerbated by intensifying climate change. Our results suggest that infected tadpoles engage in fine-tuned thermoregulation to balance the benefits of behavioural cooling against a pathogen that can perform well under warm conditions with the immune-suppressive and developmental costs of lower body temperatures. Understanding how temperature simultaneously affects pathogens and host physiology is key to clarifying host–pathogen dynamics and guiding amphibian conservation.

## Methods

### The study species

The agile frog (*Rana dalmatina* Fitzinger, 1838) is endemic to large parts of continental Europe and southern Scandinavia. It occupies a wide variety of aquatic and terrestrial habitats at altitudes ranging between zero to 1700 m above sea level [71]. Over much of its distribution range, it is the first anuran amphibian to start yearly breeding activities, spawning already in late winter or early spring [72, 73]. The critical thermal maximum of agile frog tadpoles raised in the wild is 36.8 °C [64], which is relatively low compared to that of other temperate amphibian larvae [74]. This indicates that the agile frog is a rather cool-adapted species.

### Animal collection and husbandry

In late March and early April 2021, we collected agile frog eggs on two occasions separated by 1 week. On both occasions, we collected approximately 100 eggs from each of five freshly laid clutches (hereafter sib-groups) from two natural populations (Nagykovácsi Békás-tó 47° 34′ 34″ N, 18° 52′ 08″ E and Lendület-tó 47° 33′ 04″ N, 18° 55′ 36″ E) located in the vicinity of Budapest, Hungary. We transported eggs to the nearby Júliannamajor Experimental Station of the Plant Protection Institute. Until hatching, we reared embryos separated by sib-groups in plastic dishpans (24 × 16 × 13 cm) holding 1 L of reconstituted soft water (RSW; 48 mg NaHCO_3_, 30 mg CaSO_4_ × 2 H2O, 61 mg MgSO_4_ × 7 H_2_O, 2 mg KCl added to 1 L reverse-osmosis filtered, UV-sterilized tap water). After hatching, each sib-group was haphazardly divided into three groups of 16 healthy larvae and placed into 15-L opaque, plastic rearing containers (37 × 27 × 16.5 cm), each holding 10 L of RSW. We changed the RSW twice a week and fed tadpoles with chopped and slightly boiled spinach ad libitum. When individuals reached developmental stage 25 (day 0) according to Gosner [75], we haphazardly selected 12 healthy tadpoles (i.e. we removed surplus individuals) from each rearing container and randomly assigned them to treatment groups (*Rv*-exposed vs. *Rv*-unexposed). Surplus individuals were released at their place of origin. On day 10, we exposed half of the tadpoles individually to *Rv* (Frog Virus 3; ATCC No. VR-567) by placing them into plastic cups holding 500 ml RSW and *Rv* at a concentration of 3 × 10^4^ plaque forming units (pfu) × ml^−1^. Experimental exposure lasted for 24 h. Simultaneously, we exposed the other half of tadpoles to a sham extract (i.e. *Rv*-free medium containing 2% foetal bovine serum) following the same procedure. Maintenance of *Rv* cultures followed earlier protocol [32]. From the arrival of eggs to the laboratory until start of the experimental trials (i.e. day 10), we maintained an air temperature of 16.09 ± 0.32 °C (mean ± SD; min: 15.66 °C; max: 17.16 °C) and the lighting was adjusted weekly to outdoor conditions, starting with 12:12 h light:dark cycles in late March, which we gradually changed to 15:9 h by the mid of May (i.e. day 21, end of experimental trials).

### Thermal environments

Due to spatial limitations in the laboratory and to increase our sample size, we performed two rounds of experimental trials consecutively, corresponding to the two cohorts of animals collected as eggs 1 week apart. We made our observation with 80 individuals in each round, using each individual once. We set up ten metal trays (206 × 72 × 13.5 cm, length × width × height, respectively) on two shelves using five columns of a laboratory shelf system, and we filled trays with tap water (Additional file 1: Fig. S3A). Along the longitudinal axis, each tray was separated into four equal compartments underwater by dividers made of 5 cm thick XPS insulation board. On top of the dividers, we fixed eight plastic half-pipes (200 × 8 × 8 cm, length × width × height, respectively) and filled them with 4.8 L of RSW, resulting in a water depth of ca. 3 cm (Additional file 1: Fig. S3B). We kept water levels in half-pipes low to minimize thermal stratification and conductive water circulation. Half-pipes were partially immersed into the surrounding water to allow for heat exchange between the water in the trays and the water in the half-pipes. Half-pipes in five trays positioned on the lower level of the shelf system (approx. 40 cm above the ground) provided homogeneously cool environments for the tadpoles, where the water temperature followed the air temperature in the laboratory. The temperatures available to tadpoles along half-pipes in the homogenously cool environment varied slightly around 18 °C (17.92 ± 0.5 °C; mean ± SD) and in the thermal gradient between 19.15 ± 1.45 °C at the cool end and 30.57 ± 1.06 °C at the warm end (for details see Additional file 1: Fig. S4). Temperatures within this range are known to influence the progression of ranavirosis [26, 29, 32] and encompass the thermal preference for agile frog tadpoles (22–24 °C; Hettyey et al., unpublished data). Moreover, during spring such water temperatures are commonly encountered by tadpoles in their natural habitats ([64]; Additional file 1: Fig. S5). To create temperature gradients in the half-pipes, we heated the external water in one end compartment of trays to ca. 40 °C with a 400-W towel dryer heating element equipped with a thermostat. In the neighbouring compartment, we raised the water temperature to 30 °C using a submersible 300-W aquarium heater (Tetra HT 300). In the third compartment, we did not modulate water temperature in trays. In the fourth compartment, we cooled the water in trays to 12 °C by circulating it through external water chillers (a Hailea HC-500A and a Hailea HC-300A connected in series). To mix the water within tray compartments, we equipped each compartment with a water pump (Tetra WP 300). To minimize the dissipation of heat through the thin walls of trays and to ensure consistent temperature conditions in half-pipes throughout the experiment, we insulated the outside (i.e. the bottom and all four sides) of the trays with XPS insulation. The trays providing homogeneously cool thermal environments for tadpoles were set up in the same way as those providing thermal gradients, except that the former lacked heating and cooling equipment. Between rounds, the half-pipes were removed, emptied, cleaned, and disinfected with 70% ethanol to avoid cross-contamination. Half-pipes were assigned randomly to *Rv* treatments.

### Experimental trials

During the trials, laboratory air temperature was 18.69 ± 1.67 °C (mean ± SD). On day 11, we measured the water temperature along the half-pipes using digital thermometers (Greisinger GTH 175/PT) at mid-depth of the water column. We took measurements at 20-cm intervals in the case of thermal gradients and at 50-cm intervals in the case of homogeneously cool environments (Additional file 1: Fig. S4). We performed temperature recordings twice before the first round and twice before the second round. After temperature measurements, we started experimental trials by placing one tadpole into the centre of each half-pipe. Over the following 5 days (days 12–16), we recorded the thermoregulatory behaviour of tadpoles by taking video recordings (30 fps) for 12 s, each followed by a 2-min break, for 3 h each day between 10 am and 1 pm, using car cameras (Concorde RoadCam HD 10) positioned above the half-pipes. This resulted in 78 video recordings per individual for each of the 5 days of observation. Following the last video recording each day, we fed each tadpole with 5 food pellets (Sera GranuGreen) placed at 20-cm intervals along the half-pipes to provide ad libitum food at any one feeding spot and thereby avoid forcing hungry tadpoles into areas they would otherwise not move to. Uneaten food pellets and excrements of tadpoles were removed every morning before the start of the first video recording to avoid confounding effects of foraging behaviour on thermoregulatory behaviour. To compensate for evaporation, after food removal we topped up water to the original level in half-pipes with reverse-osmosis filtered water of 18 °C or pre-heated to 25 °C (in the half-pipes providing a homogeneously cool environment or a thermal gradient, respectively). We finished this procedure before 9 am to allow at least 1 h for the temperature to set before starting recordings. At the end of each round (1st round: day 16 and 2nd round: day 21), we measured the body mass of tadpoles to the nearest 0.01 g using a laboratory scale (Ohaus PA114), euthanized them using the ‘cooling then freezing’ method [76], and conserved each in 3 ml of 96% ethanol.

### Video analysis

From the middle frame of the 12-s recordings, we determined the spatial position of tadpoles. This was facilitated by a scale (ticks every 10 cm) drawn along the longitudinal axis of half-pipes. We estimated the temperature of the immediate thermal environment of the animals by projecting their spatial position (estimated to the nearest cm) on half-pipe specific maps of water temperatures. By averaging measurements across the two rounds, we generated half-pipe–specific water temperature maps, assuming quadratic plane-curve changes in temperature between adjacent points. In accordance with previous similar studies [77], we assumed that the body temperature of a given tadpole corresponded well with the temperature of its immediate thermal environment, justified by the small size of the tadpoles and the resulting negligible thermal inertia. We used these estimated temperature values as proxy for the preferred body temperatures (T_pref_) of tadpoles that were allowed to thermoregulate, and we calculated the interquartile range of temperatures selected over a day (T_IQR_; equivalent to the width of the set-point range or T_set_ in the thermal ecology literature) [1, 2]. Activity was estimated by measuring the distances between consecutive positions of tadpoles determined from recordings taken 2 min apart.

### Molecular analysis

To assess *Rv* infection intensity, we excised the livers and homogenized them with a disposable pellet mixer (VWR, catalogue no. 47747–370). We extracted DNA with Wizard Genomic DNA Purification Kits (Promega, Madison, WI, USA) according to the manufacturer’s protocol. We stored extracted DNA at − 20 °C until further analysis. We estimated *Rv* infection intensities by a qPCR assay targeting the major capsid protein (MCP) gene of the virus following standard amplification methodologies [78]. We used the following primer sequences (5′–3′) RanaF1: CCAGCCTGGTGTACGAAAACA and RanaR1: ACTGGGATGGAGGTGGCATA and fluorescent-labelled probe (5′–3′) RanaP1: /FAM/ TGGGAGTCGAGTACTAC /MGBEQ/ for the amplification. Dilutions of purified MCP amplicons were used for setting the standard curves and these had been correlated to infective virion titres in the same qPCR as detailed earlier [32]. We ran samples in duplicate, and when the result was equivocal, we repeated reactions in duplicate. If we obtained an equivocal result again, we considered the sample *Rv* positive. Reactions were run on a BioRad CFX96 Touch Real-Time PCR System.

### Statistical analysis

We created three categories of experimental animals according to their *Rv*-status, based on combinations of *Rv*-exposure history and infection status at the end of the experiment: (1) Rv-exposed and *Rv*-positive at termination (*Rv*^+^ henceforth), (2) *Rv*-exposed, but *Rv*-negative at termination (*Rv*^−^ henceforth), and (3) not exposed to *Rv* (*Rv*^0^ henceforth). We treated *Rv*^−^ individuals as a separate group because it remains unclear whether these *Rv*^−^ individuals were not successfully infected or if they cleared the infection during the experiment.

Mortality during the experiment was very low, with only a single *Rv*^0^ tadpole dying in the first round and a single *Rv*-exposed tadpole dying in the second round of trials. We found two individuals in the *Rv*^0^ group that were cross-contaminated with the virus, but infection intensities were very low in these cases (18 and 48 pfu ml^−1^, respectively). These individuals were excluded from the analyses. This resulted in the following sample sizes: 38 *Rv*^0^, 11 *Rv*^−^, and 29 *Rv*^+^ animals in the homogeneously cool environment and 39 *Rv*^0^, 11 *Rv*^−^, and 28 *Rv*^+^ animals in the thermal gradients.

First, we analysed the relationship of T_pref_ and T_IQR_ with *Rv*-status focusing on tadpoles kept in the thermal gradients. To estimate T_pref_, we entered all temperature records of each individual as repeated measures in a linear mixed model (LMM). For estimating T_IQR_, we calculated the interquartile range of selected temperatures for each of the 5 days for each tadpole and used these 5 values as repeated measures in an LMM. In both models, we included the round of the experiment (i.e. first vs. second round) as a fixed factor, while individual ID nested in sib-group and half-pipe ID were entered as random factors. To allow for differences in the slope of temporal change of thermoregulatory behaviour amongst the three treatment groups, in both models we included the interaction between *Rv*-status (fixed factor) and the number of days since exposure treated as a covariate. Adding within-day autocorrelation into the model of T_pref_ did not change our results qualitatively; therefore, we are only presenting the results of the simpler model (without within-day autocorrelation).

Second, we analysed the activity of all experimental animals by entering the distances between consecutive positions of each tadpole as repeated measures in a generalized linear mixed model (GLMM) with negative-binomial error. As fixed effects, the model included the type of thermal environment (i.e. homogeneously cool vs. gradient), *Rv*-status, the number of days since exposure, all their two-way interactions and their three-way interaction, and the round of the experiment. As random factors, we used the same random structure as for T_pref_ and T_IQR_.

Third, because *Rv* infection intensity was measured on the 5th day of observation, we used the behavioural data of that day to analyse the effects of infection intensity on T_pref_ and activity within *Rv*^+^ individuals. These models had the same structure as above, but the fixed effects were infection intensity (ln-transformed for better model fit), round of the experiment, thermal environment, and its interaction with infection intensity.

To compare the temperatures selected by tadpoles between thermal gradients and homogeneously cool environments, we used an LMM including the round of experiment as an additional fixed factor and individual nested in sib-group as a random factor. We allowed for different variance in the two types of thermal environment. Third, we tested the effects of the thermal environment on *Rv* prevalence, as well as on infection intensity amongst *Rv*^+^ using GLMMs, with binomial and negative-binomial error, respectively. The type of thermal environment and the round of the experiment were included as fixed factors, while half-pipe ID and sib-group were used as crossed random factors.

All analyses were run in R 4.3.2 [79], using the packages ‘*lme4*’ [80] (functions ‘*glmer*’ and ‘*lmer*’) for binomial and linear models, ‘*nlme*’ (‘*lme*’ and ‘*varIdent*’ functions) for the linear model with group-specific variances, ‘*glmmTMB*’ [81] for negative-binomial models, ‘car’ (function ‘*Anova*’) for analysis-of-deviance tables (Wald *χ*^2^ test), ‘*emmeans*’ [82] for calculating marginal means and linear contrasts, and ‘*DHARMa*’ [83] for residual diagnostics. All our statistical tests had 95% confidence level. We used the false discovery rate (FDR) method to correct *P* values for multiple comparisons across *Rv*-status groups and days [84]. To illustrate significant differences in graphs, we present the model-derived mean estimates with 84% confidence intervals (CIs), because the lack of overlap between two such intervals is equivalent to a significant difference at 5% significance level [85, 86]. Note that unlike family-wise error corrections such as Bonferroni adjustment, FDR procedures do not have a simple corresponding adjustment of ordinary confidence intervals [87].

## Supplementary Information

Additional file 1: Tables S1 and S2. Table S1 Behavioural changes with time in each treatment group. Table S2 Pairwise comparisons of the slopes of behavioural changes over time. Figures S1–S5. Fig. S1 Distribution of tadpole temperatures and *Ranavirus* infection intensities in the two thermal environments. Fig. S2 Distribution of tadpole body mass at the end of behavioural observations. Fig. S3 The setups for providing different thermal environments in this study. Fig. S4 Temperatures measured along half-pipes in the homogeneous and gradient thermal treatments when no tadpoles were present. Fig. S5 Distribution of water temperatures measured in May 2022 in ponds where agile frog tadpoles developed.

## Data Availability

The dataset analysed during the current study are available in the Figshare Digital Repository, [doi: https://doi.org/10.6084/m9.figshare.30273211].

## Abbreviations

CI: Confidence interval
FDR: False discovery rate
FV3: Frog Virus 3
GLMM: Generalized linear mixed model
LMM: Linear mixed model
MCP: Major capsid protein
*Rv*: *Ranavirus*
*Rv*^−^: *Ranavirus*-Exposed and *Ranavirus*-negative tadpole at termination
*Rv*^+^: *Ranavirus*-Exposed and *Ranavirus*-positive tadpole at termination
*Rv*^0^: *Ranavirus*-Unexposed tadpole
T_IQR_: Interquartile range of daily selected temperatures over a day
T_pref_: Preferred body temperature
