# Supplementary material for: Experimental evidence for behavioural cooling as a response to virus infection in an ectothermic vertebrate
Source: BMC Biol. 2026 Jul 2;24:206. doi: 10.1186/s12915-026-02669-6 (PMC13632099; doi:10.1186/s12915-026-02669-6)

Table S1. Behavioural changes with time (per day over 5 days) in each treatment group. Estimated marginal means of linear trends (slopes) from statistical models, associated standard errors (SE) and lower (LCL) and upper (UCL) 95% confidence limits, in each infection-status group are shown for temperature preference (T_{pref}) and interquartile range of selected temperatures over a day (T_{IQR}) in thermal gradients, and activity in both thermal environments. Slopes highlighted in bold differ from zero at a 5% significance level. For comparisons of slopes to each other, see Supplementary Table S2.

Dependent variable	Slope	SE	LCL	UCL
**T_{pref} in thermal gradient**				
Rv^0	**-0.235**	0.0149	-0.264	-0.206
Rv^-	**-0.217**	0.0278	-0.271	-0.162
Rv^+	**-0.553**	0.0175	-0.587	-0.518
**T_{IQR} in thermal gradient**				
Rv^0	0.051	0.085	-0.116	0.219
Rv^-	0.140	0.157	-0.170	0.451
Rv^+	**-0.231**	0.099	-0.428	-0.036
**Activity in homogeneously cool environment**				
Rv^0	0.010	0.006	-0.001	0.022
Rv^-	0.121	0.012	0.097	0.144
Rv^+	**-0.071**	0.007	-0.085	-0.057
**Activity in thermal gradient**				
Rv^0	0.02	0.006	0.008	0.032
Rv^-	0.081	0.012	0.057	0.106
Rv^+	**-0.019**	0.007	-0.033	-0.005

Rv^0: *Rv*-unexposed; Rv^-: *R*v-exposed but *Rv*-negative; Rv^+: *Rv*-exposed and *Rv*-positive

Table S2. Pairwise comparisons of the slopes of behavioural changes over time. The infection-status groups are compared to each other in the thermal gradient for temperature preference (T_{pref}) and the interquartile range of selected temperatures over a day (T_{IQR}). Additionally, pairwise comparisons of infection-status groups for activity levels in both thermal environments are provided. Linear contrasts (*c*), associated standard errors (SE), and *P*-values adjusted using the FDR method are reported. Significant differences (P < 0.05) are in bold.

Dependent variable	Pairwise comparison	*c*	SE	*P*
**T_{pref} in thermal gradient**				
	Rv^0 *vs*. Rv^-	-0.018	0.031	0.559
	Rv^0 vs. Rv^+	0.317	0.023	< **0.001**
	Rv^- vs. Rv^+	0.335	0.032	< **0.001**
**T_{IQR} in thermal gradient**				
	Rv^0 *vs*. Rv^-	-0.089	0.179	0.618
	Rv^0 *vs*. Rv^+	0.283	0.131	0.069
	Rv^- *vs*. Rv^+	0.372	0.186	0.069
**Activity**				
	Rv^0 hom. - Rv^- hom.	-0.11	0.014	< **0.001**
	Rv^0 hom. - Rv^+ hom.	0.083	0.01	< **0.001**
	Rv^0 hom. - Rv^0 gradient	-0.01	0.009	0.252
	Rv^0 hom. - Rv^- gradient	-0.071	0.014	< **0.001**
	Rv^0 hom - Rv^+ gradient	0.031	0.01	**0.001**
	Rv^- hom - Rv^+ hom	0.193	0.014	< **0.001**
	Rv^- hom - Rv^0 gradient	0.10	0.014	< **0.001**
	Rv^- hom - Rv^- gradient	0.039	0.017	**0.025**
	Rv^- hom. - Rv^+ gradient	0.141	0.014	< **0.001**

Rv^{+} hom. - *Rv*^{0} gradient	-0.093	0.009	< **0.001**
Rv^{+} hom. - *Rv*$^{-}$ gradient	-0.153	0.014	< **0.001**
Rv^{+} hom. - *Rv*$^{+}$ gradient	-0.052	0.01	< **0.001**
Rv^{0} gradient - *Rv*$^{-}$ gradient	-0.061	0.014	< **0.001**
Rv^{0} gradient - *Rv*$^{+}$ gradient	0.041	0.009	< **0.001**
Rv^{-} gradient - *Rv*$^{+}$ gradient	0.102	0.014	< **0.001**
Rv^{+} hom. - *Rv*$^{+}$ gradient	-0.052	0.01	< **0.001**
Rv^{0} gradient - *Rv*$^{-}$ gradient	-0.061	0.014	< **0.001**
Rv^{0} gradient - *Rv*$^{+}$ gradient	0.041	0.009	< **0.001**
Rv^{-} gradient - *Rv*$^{+}$ gradient	0.101	0.014	< **0.001**

Rv^{0}: *Rv*-unexposed; *Rv*$^{-}$: *R*v-exposed but *Rv*-negative; *Rv*$^{+}$: *Rv*-exposed and *Rv*-positive; hom: Homogenously cool environment; gradient: thermal gradient

Fig. S1. Distribution of A) tadpole temperatures and B) *Ranavirus* (*Rv*) infection intensities in the two thermal environments. In each violin plot, the black dot and the white box represent the median and interquartile range, whiskers extend to 1.5 × interquartile range, and the grey polygon is a kernel density plot.

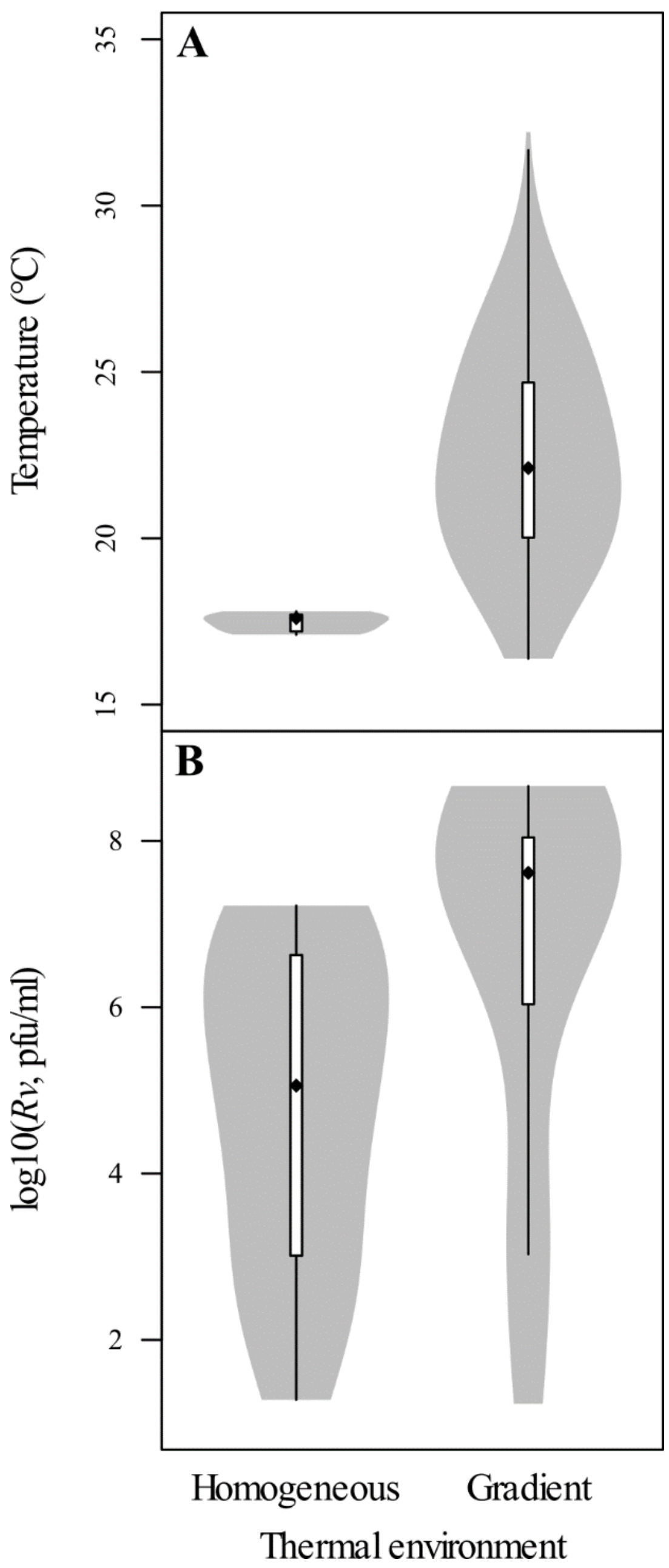

Fig. S2. Distribution of tadpole body mass at the end of behavioural observations. In each violin plot, the black dot and the white box represent the median and interquartile range, whiskers extend to 1.5 × interquartile range, and the grey polygon is a kernel density plot.

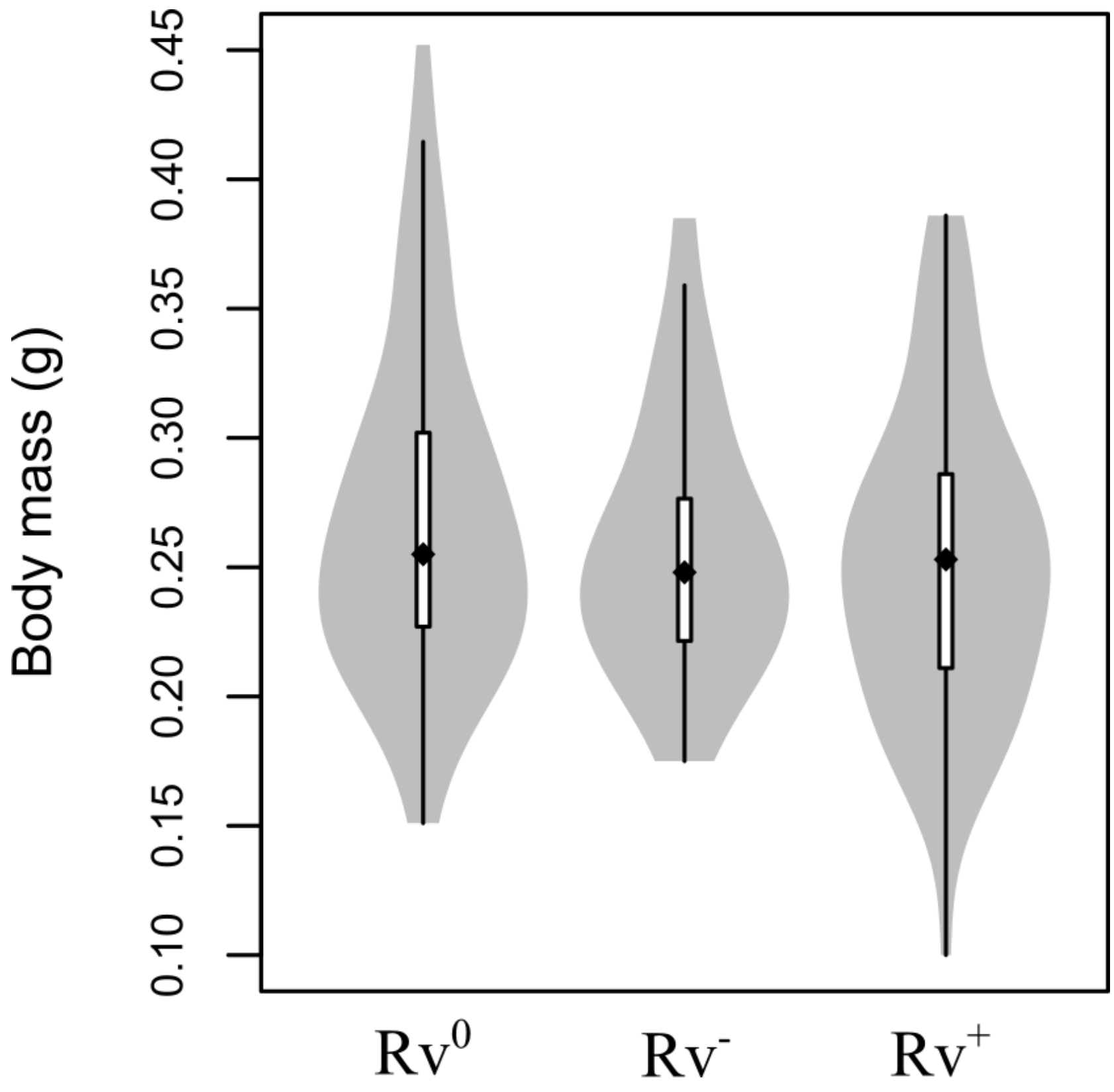

Fig. S3. The setups for providing different thermal environments in this study. (A) Half-pipes located on the upper level of the shelf system, creating thermal gradients and (B) half-pipes positioned on the lower level, providing homogeneously cool environments. Components: 1 – 400 W towel dryer heating element equipped with a thermostat; 2 – Warm end of insulated metal tray; 3 – Warm end of half-pipes; 4 – One of three dividers forming four compartments in the metal tray; 5 – Cool end of half-pipes; 6 – Tubes for cold water circulation (supplied by external water chillers) at the cool end of the tray; 7 – Car cameras used for behavioural recording; 8 – Agile frog tadpole in the homogeneously cool environment.

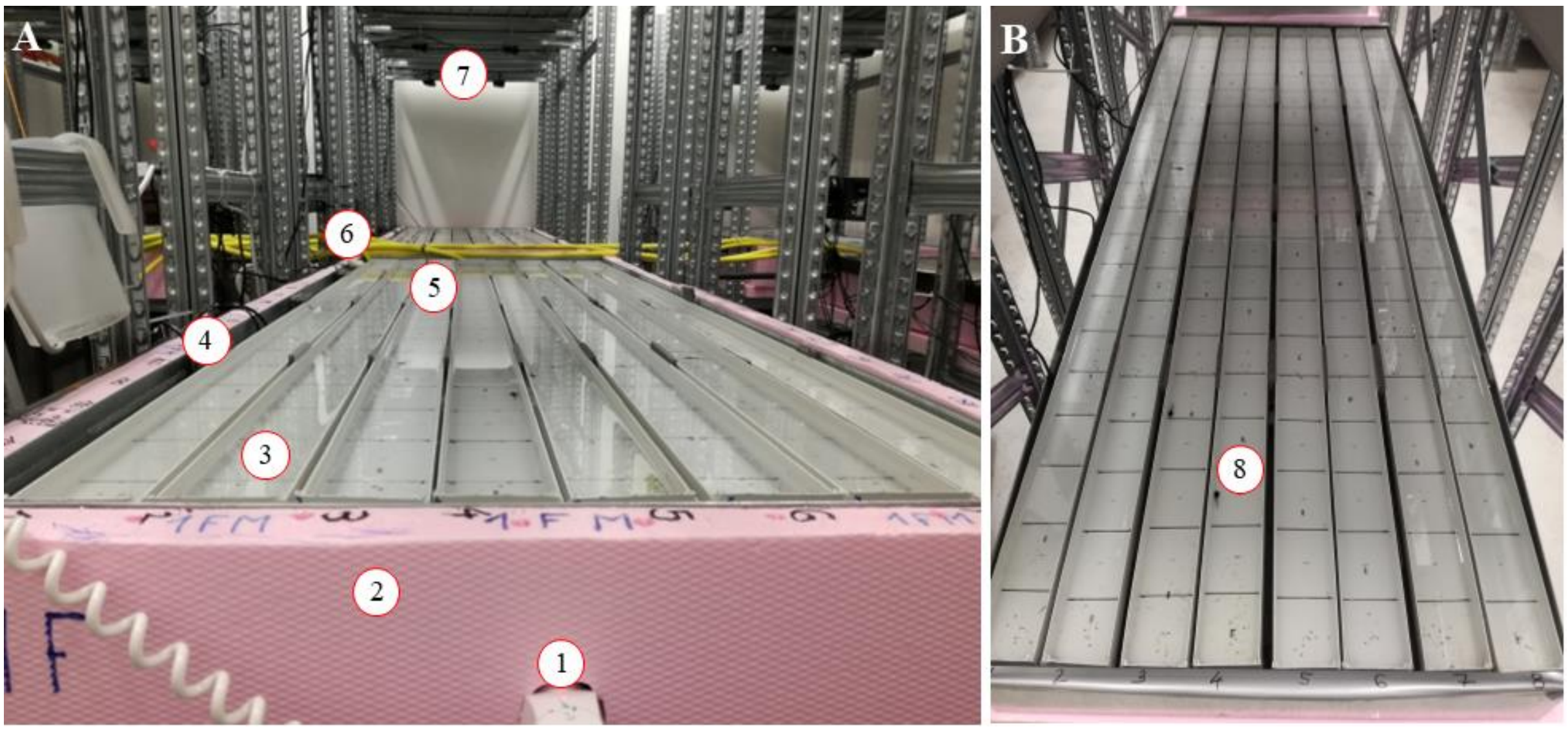

Fig. S4. Temperatures measured along half-pipes in the homogeneous (white boxes) and gradient (grey boxes) thermal treatments when no tadpoles were present. Boxplots show the median (think black line) and interquartile range (box), whiskers extend to the minimum and maximum. For each boxplot, the sample size is 160 measurements (each one of 40 half-pipes measured 4 times). The thinner and wider grey stripes, respectively, represent the 95% confidence interval and prediction interval of the linear regression fitted on the data.

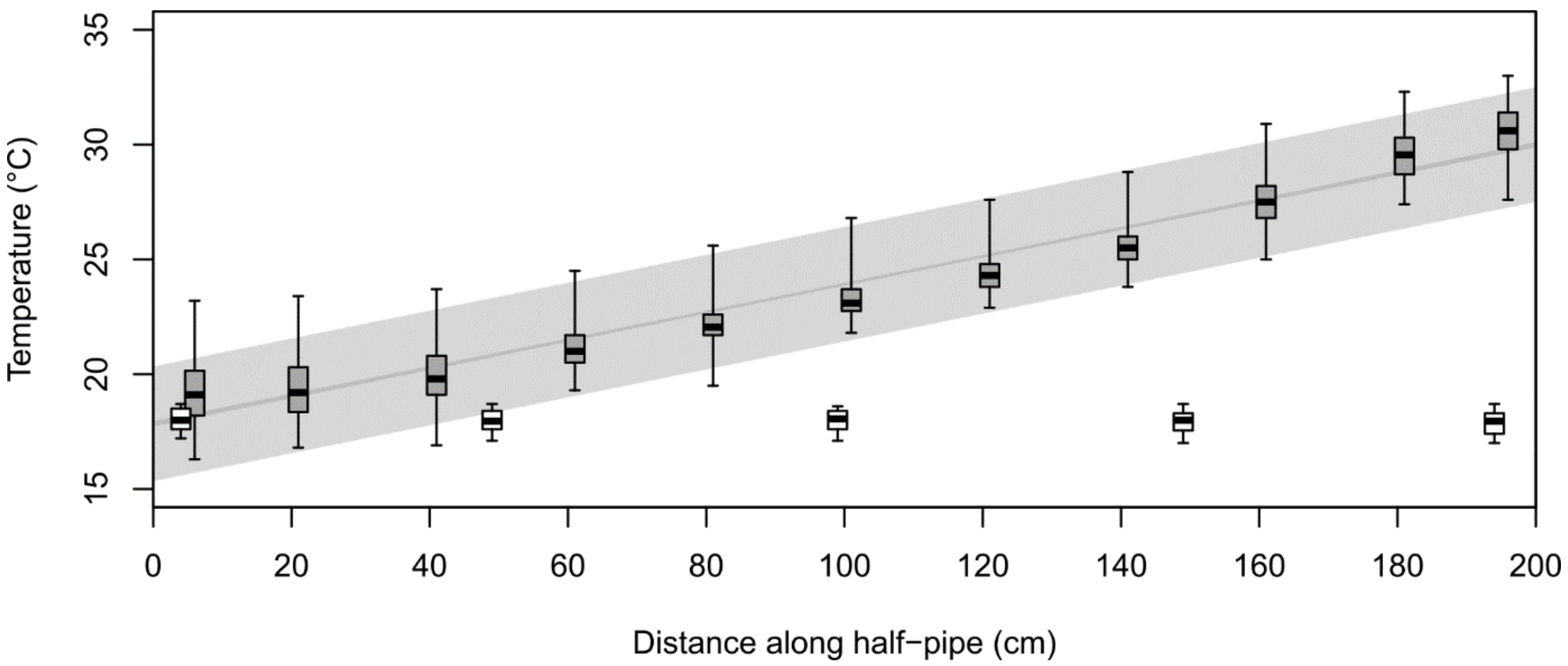

Fig. S5. Distribution of water temperatures measured in May 2022 in ponds where agile frog tadpoles developed (taken from reference 61). In the violin plot, the black dot and the white box represent the median and interquartile range, whiskers extend to 1.5 × interquartile range, and the grey polygon is a kernel density plot.

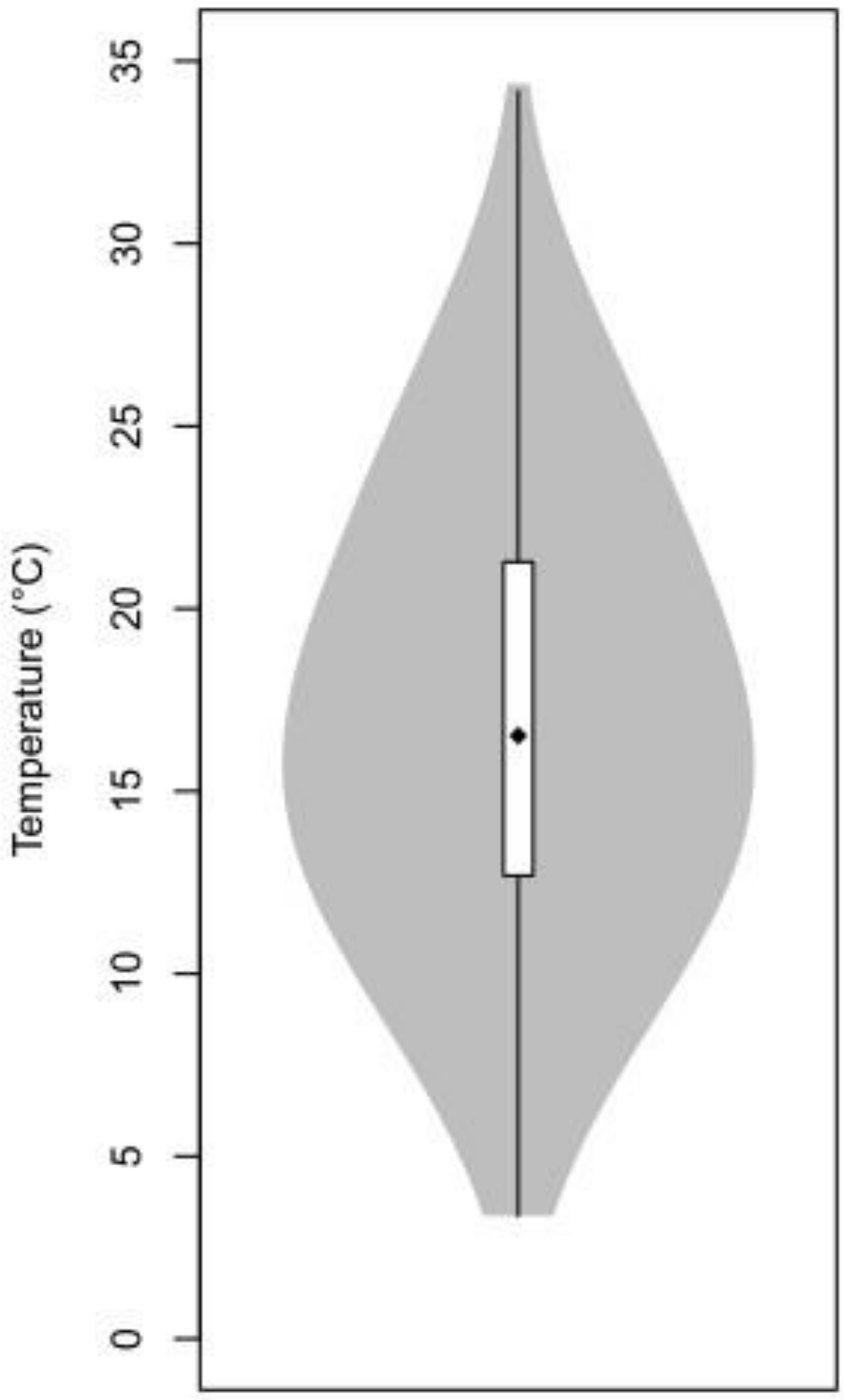

Supplement: Supplementary file 1 [file 12915_2026_2669_MOESM1_ESM.pdf]
